# Body Protein Sparing in Hibernators: A Source for Biomedical Innovation

**DOI:** 10.3389/fphys.2021.634953

**Published:** 2021-02-18

**Authors:** Fabrice Bertile, Caroline Habold, Yvon Le Maho, Sylvain Giroud

**Affiliations:** ^1^University of Strasbourg, CNRS, IPHC UMR 7178, Laboratoire de Spectrométrie de Masse Bio-Organique, Strasbourg, France; ^2^University of Strasbourg, CNRS, IPHC UMR 7178, Ecology, Physiology & Ethology Department, Strasbourg, France; ^3^Centre Scientifique de Monaco, Monaco, Monaco; ^4^Research Institute of Wildlife Ecology, Department of Interdisciplinary Life Sciences, University of Veterinary Medicine Vienna, Vienna, Austria

**Keywords:** hibernation, fasting, lean mass, metabolic depression, muscles, obesity, biomimicry

## Abstract

Proteins are not only the major structural components of living cells but also ensure essential physiological functions within the organism. Any change in protein abundance and/or structure is at risk for the proper body functioning and/or survival of organisms. Death following starvation is attributed to a loss of about half of total body proteins, and body protein loss induced by muscle disuse is responsible for major metabolic disorders in immobilized patients, and sedentary or elderly people. Basic knowledge of the molecular and cellular mechanisms that control proteostasis is continuously growing. Yet, finding and developing efficient treatments to limit body/muscle protein loss in humans remain a medical challenge, physical exercise and nutritional programs managing to only partially compensate for it. This is notably a major challenge for the treatment of obesity, where therapies should promote fat loss while preserving body proteins. In this context, hibernating species preserve their lean body mass, including muscles, despite total physical inactivity and low energy consumption during torpor, a state of drastic reduction in metabolic rate associated with a more or less pronounced hypothermia. The present review introduces metabolic, physiological, and behavioral adaptations, e.g., energetics, body temperature, and nutrition, of the torpor or hibernation phenotype from small to large mammals. Hibernating strategies could be linked to allometry aspects, the need for periodic rewarming from torpor, and/or the ability of animals to fast for more or less time, thus determining the capacity of individuals to save proteins. Both fat- and food-storing hibernators rely mostly on their body fat reserves during the torpid state, while minimizing body protein utilization. A number of them may also replenish lost proteins during arousals by consuming food. The review takes stock of the physiological, molecular, and cellular mechanisms that promote body protein and muscle sparing during the inactive state of hibernation. Finally, the review outlines how the detailed understanding of these mechanisms at play in various hibernators is expected to provide innovative solutions to fight human muscle atrophy, to better help the management of obese patients, or to improve the *ex vivo* preservation of organs.

## Introduction

The maintenance of a stable body composition is essential to ensure overall health and performance. Each organism can roughly be separated into a fat and a lean compartment, healthy proportions in humans ranging from 12 to 30% of fat and 70 to 88% of fat-free mass (Abernathy and Black, 1996). The lean compartment is composed of mainly water (73%) and proteins (20%) (Wagner and Heyward, 2000), and excessive loss of lean body or protein mass has been associated with a myriad of adverse effects (Willoughby et al., 2018). It is estimated that the loss of 30–50% of total body proteins is directly responsible for death (Silber, 1984).

Although ubiquitous in the body, proteins are predominantly found in muscles, which in humans represent ~40% of total body weight (Janssen et al., 2000). Besides their obvious importance for posture maintenance, locomotion, and any movement from the cellular scale to the whole body, muscles are nowadays regarded as important modulators of the whole-body energy metabolism and interorgan cross-talks (Argiles et al., 2016). Muscles ensure organs to be continuously supplied with oxygen and nutrients while waste products are excreted not only by supporting the respiratory and circulatory systems but also through their endocrine/paracrine functions (Karstoft and Pedersen, 2016; Giudice and Taylor, 2017; Graf and Ferrari, 2019). Muscles are important consumers of lipids and carbohydrates and store glycogen to be mobilized in case of reduced glucose availability in food supply (Frontera and Ochala, 2015; Argiles et al., 2016). Further, the high protein content of muscles makes up a large reservoir of amino acids for protein synthesis within the body (Wolfe, 2006; Argiles et al., 2016) and for hepatic gluconeogenesis during starvation (Ruderman, 1975; Argiles et al., 2016). Finally, muscle shivering or non-shivering thermogenesis has also been involved in tuning body temperature (T_b_) and energy expenditure, with an ultimate control on body weight (Periasamy et al., 2017; Fuller-Jackson and Henry, 2018). Given such essential roles, it is not surprising that metabolic health relies on the maintenance of muscle structure and function (Hunt, 2003; Wolfe, 2006; McLeod et al., 2016; Deutz et al., 2019).

Muscle atrophy, or muscle wasting, can be defined as a loss of muscle mass, strength, and mobility. Causes of muscle atrophy in humans are multiple and notably include aging (McCormick and Vasilaki, 2018), malnutrition (Roy et al., 2016), prolonged fasting (Ibrahim et al., 2020b), disuse observed in immobilized patients or due to sedentary lifestyles (Rudrappa et al., 2016), denervation (Carlson, 2014), microgravity environments (Gao et al., 2018), and a variety of diseases (Schardong et al., 2018; Sisto et al., 2018; Song et al., 2018; Yang et al., 2018; Zhang et al., 2018). Contrary to humans, hibernating animals usually lose no or very little muscle mass during winter in metabolic depression, despite several months of complete food deprivation and physical inactivity after a nearly doubling level of fat mass (see below). In this review, a brief description of the main features of torpor and hibernation is followed by a detailed compilation of the available data on the preservation of body and skeletal muscle proteins during hibernation. Then, it elaborates on the various mechanisms that may help sustain protein homeostasis, involving the mechanisms of metabolic rate depression, muscle shivering, urea and nitrogen recycling, the role played by a number of humoral factors, and the regulation of intracellular pathways. Finally, this review also presents how the outstanding performances of hibernators can very likely fuel innovative solutions for humans to fight muscle atrophy and promote therapies for preserving body proteins.

## Maintenance of Lean Body Mass in Hibernators During Winter

### Torpor and Hibernation

Torpor is an energy-saving strategy used by small heterothermic mammals and birds, involving a controlled reduction of metabolic rate (MR) and T_b_, which enables animals to survive periods of energetic bottleneck (Lyman et al., 1982). Heterothermic species can be differentiated in the so-called “daily heterotherms,” i.e., species undergoing rather shallow (12–25°C) bouts of torpor of less than 24 h, and “hibernators” that undergo long and deep (<10°C) bouts of torpor lasting for days or weeks (Ruf and Geiser, 2015). Hibernation is documented in mammals from all three subclasses but is known for only one bird species (Ruf and Geiser, 2015). It is often associated with species inhabiting cold and seasonal habitats, such as temperate and arctic zones, but it is also used by many non-Holarctic species, i.e., in the tropics and southern hemisphere (Nowack et al., 2020). During hibernation, animals reach minimum torpid MR of ~ 4% of basal MR, in association with a more or less pronounced reduction of their T_b_ ranging on average for most hibernators between 0°C and 10°C (Ruf and Geiser, 2015). However, the occurrence of subfreezing T_b_ has been documented in some hibernating species, e.g., in Arctic ground squirrels (*Urocitellus parryii*), which allow their peripheral T_b_ to drop to −2.9°C (Barnes, 1989).

In most species, hibernation is structured by successive torpor bouts and periodic interbout arousals with euthermia (Twente et al., 1977; French, 1982, 1985; Barnes et al., 1986; Carey et al., 2003b). In small mammals, MR increases drastically during arousals and T_b_ returns to normothermic levels of about 35–37°C for a few hours (Carey et al., 2003a; Heldmaier et al., 2004). These arousals represent the highest proportion of energy expended during the hibernation process, e.g., 70–80% in temperate species (Wang, 1978). In Arctic ground squirrels (*U. parryii*) hibernating at 2°C, arousal episodes can even account for up to 86% of the estimated energetic costs during the hibernation season (Karpovich et al., 2009). A few hibernating species do not fatten prior to winter and must therefore feed during these arousal phases (Humphries et al., 2003b). However, for the others, i.e., the majority of hibernating species, that fast throughout hibernation, the exact purposes of these periodic arousals remain a mystery. Many hypotheses have been put forward, some of which have fairly convincing experimental data: a restoration of metabolic homeostasis (Osborne and Hashimoto, 2003; Epperson et al., 2011), a prevention of excessive accumulation of oxidative damage during torpor and arousal bouts (Buzadzic et al., 1990), the elimination of metabolic waste by renal function (e.g., Clausen and Storesund, 1971), the replenishment of carbohydrate supplies (Wang, 1989), the restoration of functional protein pools (Carey et al., 2003a), the reactivation of immune function (Prendergast et al., 2002), a pH regulation linked to the accumulation of carbon dioxide (CO_2_) (Malan et al., 1988), or to allow animals to sleep (Daan et al., 1991). However, for the latter hypothesis, accumulation of sleep need during torpor bouts has been challenged by several studies. It was notably shown that sleep deprivation during the first few hours of euthermia following arousal in ground squirrels resulted in the disappearance of the classically observed peak level of slow-wave activity at this stage, without any compensatory rebound when sleep deprivation was terminated (Larkin and Heller, 1996, 1999; Strijkstra and Daan, 1998). Hence, the early peak of slow-wave activity during arousals does not appear as a homeostatic response to an accumulated sleep debt. Similarly, the reactivation of the immune function may not be a purpose of torpor arousals, but secondary to rewarming. During the rewarming process of arousals, non-shivering thermogenesis from brown adipose tissue (BAT) is crucial until T_b_ reaches 15°C. Then, the rewarming continues *via* muscle shivering thermogenesis until the muscles are warm enough (Hashimoto et al., 2002). In the case of some tropical and subtropical species, rewarming is generally diurnal and thus passive with rising ambient temperatures (T_a_), then becoming active *via* the heat production from BAT (Geiser and Drury, 2003). Non-shivering thermogenesis can also occur within muscles *via* futile cycles of calcium (Rowland et al., 2015) and has been postulated as an alternative mechanism for heat production in all those heterotherms that completely lack BAT, e.g., heterothermic marsupials and birds (Nowack et al., 2017). Because skeletal muscle accounts for approximately 40% of the dry mass of the typical mammalian body, downregulating skeletal muscle non-shivering thermogenesis would allow for whole-body cooling and long-term maintenance of a depressed core T_b_ during the steady state of torpor. In their review dealing with thermoregulation in hibernating mammals, Oliver et al. (2019) notably highlighted the importance of skeletal muscle and sarcolipin, a peptide regulating sarcoplasmic/endoplasmic reticulum Ca^2+^ ATPase (SERCA) activity, as a major thermogenic target.

In few other heterotherms, hibernation constitutes a continuous torpid state, showing a lack of arousals in, e.g., hibernating *Ursus arctos* (Evans et al., 2016), *Tenrec ecaudatus* (Lovegrove et al., 2014), and free-ranging *Cheirogaleus medius* (Dausmann et al., 2005), the latter nevertheless possibly having multiday torpor bouts interrupted with metabolic heat production when hibernating in well-insulated tree holes (Dausmann et al., 2004). Historically, due to a definition based not only on metabolic depression but also on characteristics that are secondary to it (Watts et al., 1981), mammalian hibernation has been restrictively attributed to only those animal species of less than 5–10 kg for which not only MR is reduced by 90–95% during the hibernation period but also T_b_ is lowered below 10°C (Nedergaard and Cannon, 1990; Geiser, 2011). One of the main reasons resides in the allometric scaling of MR with body mass, which has been greatly discussed elsewhere (Geiser, 2004; Heldmaier et al., 2004; Staples, 2016). Briefly, larger animals have lower basal MR per unit of body mass than smaller ones. Animals' surface area-to-volume ratio may contribute to explain this difference, larger animals having less body surface—across which heat is exchanged with the environment—relative to their volume, representing the amount of tissue that produces heat *via* metabolism. As a result, larger animals, with low surface area-to-volume ratios, lose less heat than smaller animals in a cool environment, and less energy, i.e., a lower MR, is needed to maintain T_b_. In addition, if an important cooling would occur in large animals, a huge amount of energy would be required thereafter for body rewarming, which could not be achievable in a hibernation context. Therefore, hibernation could be expected to be of lower advantage for larger animals in terms of energy savings. Today, we know that large animals, such as bears (family *Ursidae*), undergo hibernation as they exhibit features of metabolic depression very similar to those found in small hibernators (Staples, 2014). Similar values of minimal specific MR around 0.03 ml O_2_.g^−1^.h^−1^ are in fact observed in all small and large hibernators during the hibernation period (Heldmaier et al., 2004), thus indicating that the allometric scaling of MR with body mass is disrupted during torpor. Similar values are also observed in the largest animals on Earth, elephants and blue whales (Singer, 2006). As suggested earlier, minimal specific MR reached during hibernation may therefore represent a lower limit to ensure that cell viability is maintained. With a specific MR similarly lowered in small and large hibernators, it has been calculated that the low surface area-to-volume ratio of large hibernators implies a limited drop in T_b_, such as of a few degrees Celsius only for bears (Hochachka and Guppy, 1987). Accordingly, hibernating bears exhibit a 75–85% decline in MR but a T_b_ decreased by only few degrees Celsius compared to values during the summer-active season, hence remaining at around 32–33°C (Watts et al., 1981; Hissa et al., 1994; Toien et al., 2011; Evans et al., 2016). Thermoregulatory mechanisms could explain the maintenance of a relatively high T_b_ in bears. Indeed, previous studies have revealed that hibernating grizzly (*U. arctos horribilis*) and polar bears (*U. maritimus*) still express a circadian rhythm in locomotor activity (Jansen et al., 2016; Ware et al., 2020), whereas other reports showed that black bears (*U. americanus*) replace their circadian rhythm of activity and body temperature by multi-day cycle during hibernation (Toien et al., 2011). In addition to reflecting putative thermoregulatory mechanisms, multi-day cycles may suggest a unique capacity to slow down MRs and ultimately biological time (Malan et al., 2018). Not only changes in body surface temperature have suggested that black bears engage in bouts of muscle activity during hibernation (Harlow et al., 2004), but bursts of shivering have also directly been measured in another study (Toien et al., 2011). The muscles from captive grizzly bears have also been reported to shudder during hibernation for periods lasting greater than 1 h (Lin et al., 2004). However, thermoregulation may not be of major importance to support hibernation at only mild hypothermia in bears. First, increased body insulation in hibernating bears due to important fur covering (Scholander et al., 1950b) and subcutaneous fat accumulation (Svihla and Bowman, 1954) is expected to drastically lower heat loss. Second, T_a_ in the dens of Colorado black bears (*Ursus americanus*) has been reported to be +10°C on average despite outside T_a_ fluctuating from −20°C to +5°C (Harlow et al., 2004), and similar values have been reported for black bears in Canada (Watts et al., 1981). Comparable climate conditions have been reported for Scandinavian brown bears (*U. arctos*) (Evans et al., 2016). Hence, since the manipulation of den temperature in black bears (*U. americanus*) has revealed a lower critical temperature close to 0°C (Toien et al., 2015) and since a similar value has been reported for polar bears (*Ursus maritimus*) (Scholander et al., 1950a), ursids are expected to hibernate under thermoneutral conditions. In addition, it is noteworthy that, although one study has reported the presence of BAT in bears in the early 1990s (Davis et al., 1990), no BAT has later been found in bears (Jones et al., 1999; Rigano et al., 2017). Whether beige adipocytes, which also possess thermogenic properties (Ikeda et al., 2018), are present in bears is still not known.

### Energy Substrate Use During Hibernation

Most of the hibernators, i.e., fat-storing species, do not feed during hibernation and rely entirely on body fat reserves accumulated prior to hibernation (Dark, 2005). Some other hibernators, e.g., food-storing species, feed during interbout arousals and therefore hoard large amounts of food (mainly seeds) prior to winter in their burrow (French, 1988; Humphries et al., 2003b). Only a few species are food-storing hibernators, mainly hamsters and chipmunks; they undergo shorter torpor bouts than fat-storing species but have longer arousal phases during which individuals consume their food hoards (Wollnik and Schmidt, 1995; Humphries et al., 2001). Thus, fat- and food-storing species show different metabolic and digestive adaptations throughout their annual life cycle (Humphries et al., 2001, 2003a; Weitten et al., 2013; Giroud et al., 2020).

In small hibernators, the measurement of respiratory quotient (RQ) values close to 0.7 during torpor shows that the coverage of energy expenditure is provided almost exclusively by the oxidation of lipids (Kayser, 1952; Buck and Barnes, 2000). Variations in T_a_, in particular a decrease, can however trigger thermoregulatory responses (thermoregulation is not abolished during hibernation), and one can then observe higher RQ (Buck and Barnes, 2000), indicating an increase in the oxidation of carbohydrates and/or proteins. Plasma profiles during torpor are similar to those observed during fasting, with a decrease in blood glucose and triglyceride levels and an increase in blood free fatty acid and ketone body levels in most fat-storing hibernators [Belding's ground squirrels (Krilowicz, 1985), golden-mantled ground squirrels (Tashima et al., 1970; Lovegrove and McKechnie, 2008)], and food-storing species [golden hamster (Weitten et al., 2013)]. The use of proteomics has revealed changes in skeletal muscles of hibernating thirteen-lined ground squirrels, which were consistent with their reliance on lipids for energy during hibernation (Anderson et al., 2016). Measurements of the activity of key enzymes involved in metabolic pathways and of the level of gene expression during and outside the hibernation period in a number of studies have confirmed that the use of glucose is reduced in favor of that of lipids during hibernation (Dark, 2005), with the exception of glucose-dependent tissues like kidneys and brain (South and House, 1967; Rauch and Behrisch, 1981). At the beginning of the rewarming phase in small hibernators, the major substrate of BAT for non-shivering thermogenesis is still fatty acids. Then, the increase in T_b_ up to 12–16°C is accompanied by an increase in RQ to 1.0, indicating oxidation of carbohydrates, mainly by muscles for shivering thermogenesis (Mokrasch et al., 1960; Castex and Hoo-Paris, 1987; Heldmaier et al., 2004), and/or an evacuation of the CO_2_ accumulated during torpor (Malan et al., 1988). A recent study has deepened the analysis in hibernating thirteen-lined ground squirrels (Regan et al., 2019). It has shown that a combination of lipids and carbohydrates is used during the initial ~60 min of arousal before switching to predominantly lipid oxidation. To compensate for glucose utilization and to maintain glycemia during hibernation, glucose is provided by liver glycogenolysis and gluconeogenesis (Burlington and Klain, 1967; Green et al., 1984). The major gluconeogenic substrate is glycerol, whereas amino acid and lactate contribute only moderately to endogenous glucose production (Burlington and Klain, 1967; Galster and Morrison, 1975). In food-storing hibernating species, glucose is provided by food ingestion. Increased digestive efficiency and in particular upregulated intestinal glucose absorption rates during hibernation, e.g., in eastern chipmunks (Humphries et al., 2001) and common hamsters (Weitten et al., 2016), might rapidly restore blood glucose levels upon arousal phases (Serkova et al., 2007; Weitten et al., 2013).

Numerous reports have shown that black (*U. americanus*) and brown (*U. arctos*) bears remain not only physically inactive, but they also do not eat, drink, defecate, or urinate during the whole duration of the denning period (Craighead and Craighead, 1972; Nelson et al., 1975; Craighead et al., 1976; Folk et al., 1976, 1980; Hellgren et al., 1989; Hissa et al., 1994; Hellgren, 1998). Mobilization of body fuel reserves promotes survival during hibernation, essentially from body fat stored prior to denning (Nelson et al., 1975; Lundberg et al., 1976; Barboza et al., 1997). This is supported by the loss of 15–25% of bear body mass over the hibernating season (Hissa et al., 1994; Swenson et al., 2007), an RQ value close to 0.7 or slightly below (Nelson et al., 1973; Hellgren, 1998), and an increase in concentrations of circulating fatty acids (Nelson, 1973; LeBlanc et al., 2001; Chazarin et al., 2019a; Giroud et al., 2019). Plasma total ketone bodies have also been found higher in March than in June and November in Japanese black bears (Shimozuru et al., 2016), and we have observed a significant increase in 3-hydroxybutyrate in hibernating brown bears (Chazarin et al., 2019a), thus suggesting its use instead of glucose by, e.g., the brain. Liver gluconeogenesis has notably been proposed to be fueled from glycerol, which is released by white adipose cells due to lipolysis, but its plasma concentration remains unchanged due to liver uptake (Chazarin et al., 2019a). Lactate has also been suggested as a precursor for the hepatic neo-synthesis of glucose (Shimozuru et al., 2016; Chazarin et al., 2019a).

### Preservation of Body and Skeletal Muscle Proteins During Hibernation

Strikingly, black (*U. americanus*) and brown (*U. arctos*) bears exhibit no significant loss in their lean body mass during hibernation (Nelson et al., 1975; Lundberg et al., 1976; Barboza et al., 1997; Hilderbrand et al., 2000). Accordingly, whole-body protein turnover rates have been found at similar (Barboza et al., 1997) or higher (Lundberg et al., 1976) levels in bears during hibernation (winter) compared to the pre-denning period of hyperphagia (autumn), protein synthesis and breakdown being elevated in winter. Ketone body production and the preferential utilization of fatty acids and ketone bodies likely contribute to the sparing of amino acids and glucose during fasting (Owen et al., 1998). Body protein conservation is improved when lipid reserves are larger in fasting laboratory rodents (Goodman et al., 1984; Lowell and Goodman, 1987; Cherel et al., 1992), and the same mechanism may operate in hibernating bears as suggested by the fact that fatness of polar bears is inversely correlated to body protein breakdown (Atkinson et al., 1996). In this context, body protein sparing is also supported by the transcriptional downregulation of amino acid catabolism-related genes and upregulation of gluconeogenesis- and ketogenesis-related genes in the liver from hibernating Japanese black bears (*U. thibetanus japonicas*) (Shimozuru et al., 2012), American black bears (Fedorov et al., 2011), and grizzly bears (Jansen et al., 2019). Moreover, RQ values below 0.7 suggest that metabolic CO_2_ serves for anabolic processes (Nelson et al., 1973; Hellgren, 1998).

Both skeletal muscle strength and mass appear to be retained in hibernating bears. Indeed, black bears (*U. americanus*) have been shown to lose no more than 29% of tibialis anterior strength over 2 months of hibernation (Harlow et al., 2001; Lohuis et al., 2007b), while skeletal muscle cell number or size (cross-sectional area) and contractile properties remained unchanged in various skeletal muscles during hibernation (Tinker et al., 1998; Harlow et al., 2001). Two different studies have observed an increase in the proportion of fast-twitch fibers (type II) for the biceps femoris muscle from hibernating black bears, which was accompanied by decreased activity of citrate synthase, whereas no change was observed for the gastrocnemius muscle (Tinker et al., 1998; Rourke et al., 2006). In brown bears (*U. arctos*), similar results were obtained in the biceps femoris for fiber cross-sectional area, the relative proportion of fast and slow fibers, and contractile properties, which remained essentially unchanged during hibernation compared to the summer-active season (Hershey et al., 2008). Giant sarcomeric proteins (titin, nebulin) have notably been shown to be roughly maintained at normal levels during hibernation in striated muscles of brown bears (*U. arctos*) and Himalayan black bears (*Ursus thibetanus ussuricus*) (Salmov et al., 2015). Recently, a 26% decrease in sartorius muscle fiber size following hibernation has nevertheless been reported in the Japanese black bear (*Ursus thibetanus japonicus*) (Miyazaki et al., 2015). Besides the various fast muscles mentioned above, maintenance of slow oxidative soleus muscle during hibernation has been documented recently in black bears (Riley et al., 2018). It was notably shown that soleus fiber type proportions, size, average fiber cross-sectional area-to-body mass ratio, and muscle-specific tension and normalized power were not altered by the hibernating state.

Changes in protein concentrations of bear hind limb muscles may differ according to the muscle considered; they either remain stable from the period prior to hibernation to spring arousal (Koebel et al., 1991; Harlow et al., 2001) or decrease by a maximum of generally 4–10%, notably in lactating females during denning (Tinker et al., 1998; Hershey et al., 2008). One study in black bears has reported a 15% loss in the protein content of the vastus lateralis muscle after 1 month of denning, this same value having been measured again 3.5 months later (Lohuis et al., 2007a). Moreover, muscle nitrogen content was unchanged in winter compared to summer, thus indicating moderate protein loss at the transitions between the summer-active and winter-resting periods but the striking maintenance of muscle integrity over long periods of hibernation. In fact, reduced protein turnover in skeletal muscles during hibernation (decreased levels of both protein synthesis and breakdown) supports muscle preservation.

Small hibernators also spare their body proteins during hibernation, as suggested by low uremia levels [e.g., of fat-storing species: golden-mantled ground squirrels (Wit and Twente, 1983); e.g., of food-storing species: Syrian hamsters (Weitten et al., 2013)] and limited muscle atrophy (e.g., Cotton and Harlow, 2010; Nowell et al., 2011; Hindle et al., 2015). In the common hamster, a food-storing hibernator, diet quality strongly influences body composition and particularly fat-free mass during hibernation (Weitten et al., 2018). Indeed, hamsters fed a high-protein diet spent more time in torpor during hibernation than hamsters fed a high-lipid diet, thus losing less body mass and particularly no fat-free mass. The extent of muscle atrophy during hibernation is relatively small compared to inactive non-hibernating rodents, e.g., the hind limb-suspended rat model (Musacchia et al., 1988). When it occurs, atrophy develops at the beginning of hibernation in fat-storing species, regardless of T_a_ (5 or 23°C), such as, e.g., in golden-mantled ground squirrels (Steffen et al., 1991; Wickler et al., 1991; Nowell et al., 2011), and it does not progress but on the contrary, it can even disappear before the end of hibernation (Hindle et al., 2015). It is characterized by a decrease in muscle mass, i.e., muscle proteins, due mainly to a decrease in cell size and not cell number, since muscle DNA content is not altered (Steffen et al., 1991) and apoptosis is decreased (Xu et al., 2013). Even when muscle atrophy is observed during torpor, oxidative capacity is increased (Wickler et al., 1987, 1991; Steffen et al., 1991) probably because slow oxidative fibers are preserved, whereas fast-glycolytic fibers are atrophied (Hindle et al., 2015). A shift in skeletal muscle composition from fast-glycolytic to slow-oxidative fibers has also been observed (Rourke et al., 2004; Nowell et al., 2011; Gao et al., 2012). This enables heat production during rewarming from torpor and locomotor activity during interbout arousal phases and favors emergence from hibernation (Nowell et al., 2011). Contrasting with a decrease in mass of some skeletal muscles, cardiac muscle mass is increased during hibernation in golden-mantled ground squirrels (Wickler et al., 1991). In the liver also, proteolysis is reduced during hibernation (Velickovska et al., 2005).

### Metabolic Rate Depression Contributes to Protein Sparing During Hibernation

**Figure 1:**
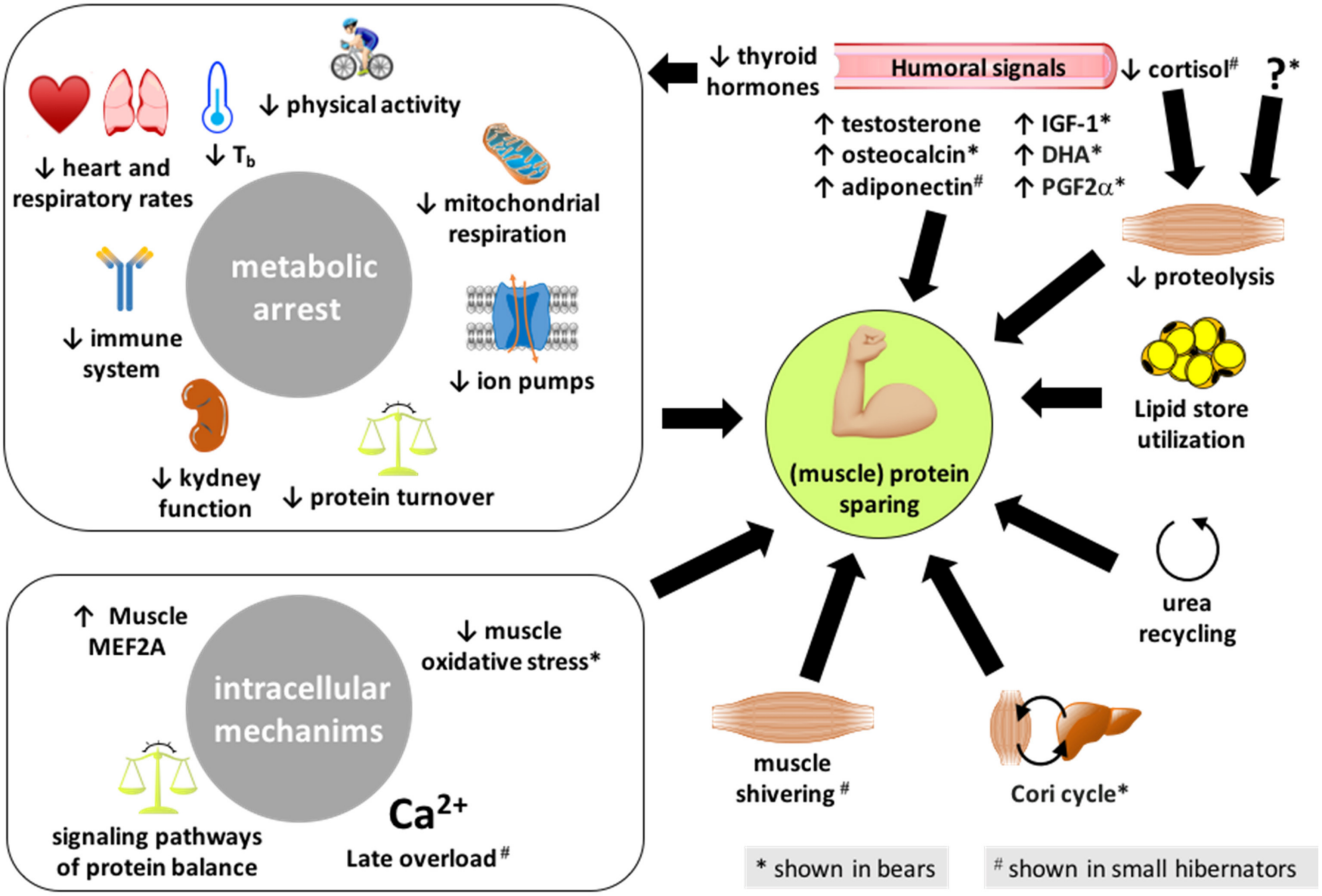
Summary of the different processes enabling hibernators to spare their (muscle) proteins. The mechanisms presented here have been reported in both bears and small mammal hibernators, except those highlighted with an * or a ^#^ that are shown or believed to be involved, respectively, in bears only or small hibernators only. IGF-1, insulin-like growth factor-1; DHA, docosahexaenoic acid; PG2α, prostaglandin F2α.

The regulatory mechanisms involved in energy savings and metabolic flexibility characterized by dropped MR during hibernation likely contribute to cell and organ preservation (Figure 1). Physiologically, energy savings during hibernation involve specific changes, i.e., reduced allocation of energy to vital and energy-costly functions such as blood circulation, respiration, digestion, immunity, and kidney waste excretion.

In black and brown bears, a significant decline in the average heart rate from 50 to 80 beats per minute during the summer-active period to 10–30 beats per minute during winter months has been reported (Folk et al., 1980; Nelson et al., 2008; Nelson and Robbins, 2010, 2015; Laske et al., 2011; Toien et al., 2011; Evans et al., 2012; Jorgensen et al., 2014, 2020). Also in bears, the respiratory rate drops from 10 to 12 breaths per minute during summer to 5–7 breaths per minute in bears during the winter (Laske et al., 2011; Evans et al., 2012). Similarly in small mammals, extremely low heart rates of 3–10 beats per minute have been recorded during hibernation compared with 200–400 beats per minute when the animals are active (Lyman et al., 1982; Hampton et al., 2010). Breathing also drops from greater than 40 to less than one breath per minute in small hibernating mammals, and breathing patterns in many species can include long periods of apnea, ranging from minutes to hours. It has been claimed that there is no gut atrophy in denning bears but a decrease in gut motility and of metabolic activity of the gut flora that may be essential to the adaptation of the bear's physiology to hibernation (Jones and Zollman, 1997). A suppression of the innate immune system has been reported in small hibernating mammals (Bouma et al., 2010, 2011) and in hibernating brown bears (*U. arctos*) (Sahdo et al., 2013; Graesli et al., 2015). However, the immune competence may be maintained in black bears (*U. americanus*) (Chow et al., 2013). Such discrepancies between bear species highlight the need for further studies to definitely conclude. At the kidney level, bear glomerular filtration rate is decreased from 117 ml per minute in the summer-active state to 37 ml per minute during bear hibernation (Brown et al., 1971), thus resulting in the production of very low amounts of urine that are reabsorbed from the bladder urothelium (Nelson et al., 1973, 1975), and hibernating bears are therefore anuric. It is noteworthy to mention that paradoxical energy-demanding processes are activated in pregnant female bears during the denning period for gestation and cub lactation (Matson, 1954; Wimsatt, 1963; Tsubota et al., 1987; Sandell, 1990; Oftedal et al., 1993; Farley and Robbins, 1995; Spady et al., 2007; Friebe et al., 2013, 2014). Nevertheless, related energetics costs are limited by a gestation period reduced to about 2 months, calving of small cubs of only 300–400 g, and a low production of fat-rich milk ranging from 2.5 to 5 g/kg.

Various mechanisms are involved in metabolic suppression, which may contribute to the sparing of (muscle) proteins during hibernation. Forty years ago, Malan (1980) highlighted that MR reduction during hibernation is likely induced by body cooling and the resulting slowing down of enzymatic rates according to the Arrhenius law, but he also stressed the superimposition of additional temperature-independent mechanisms actively contributing to hypometabolism. Since then, the majority of metabolic suppression to 25% of basal rates in hibernating black bears (*U. amercicanus*) has been reported to be independent of lowered T_b_ (Toien et al., 2011). The active mechanisms underlying metabolic depression in hibernators have been discussed several times (Carey et al., 2003a; Storey, 2010; Storey and Storey, 2010; Quinones et al., 2014; Staples, 2014, 2016; Klug and Brigham, 2015). In golden-mantled ground squirrels, liver transcription and translation are largely suspended during deep torpor and are restored upon periodic arousals (Van Breukelen and Martin, 2001; Epperson and Martin, 2002). Regulation of transcription–translation appears peculiar in bears vs. small hibernators. Liver microarray analysis in the liver from hibernating golden-mantled ground squirrel (*Spermophilus lateralis*) has revealed that 90% of differentially expressed genes are downregulated (Williams et al., 2005), whereas more balanced proportions of downregulated and upregulated genes have been reported in the liver from hibernating black bears (*U. americanus*) and protein synthesis genes were shown to be upregulated (Fedorov et al., 2009, 2011). These specific adaptive mechanisms of bear liver compared to small hibernators may be linked to hibernation at higher T_b_ and MR. It should also be noted that transcript global analysis in skeletal muscles has revealed that hibernation induces a common transcriptional programming in Arctic ground squirrels (*U. parryii*) and black bears (*U. americanus*), including coordinated induction of protein biosynthesis (translation) genes and suppression of genes involved in oxidation–reduction and glucose metabolism (Fedorov et al., 2014). In addition, protein turnover is reduced but remains at equilibrium during hibernation, both protein synthesis and breakdown rates being lower in winter compared to summer in the muscles of black bear (Lohuis et al., 2007a). Signs of reduced protein translation (Van Breukelen and Martin, 2001) and of protein catabolism have also been reported in the liver of hibernating small mammals (Velickovska et al., 2005). Reduced levels of miR-24 transcripts have been reported in heart and skeletal muscle of torpid ground squirrels as was a lowering of miR-122a levels in the muscle (Morin et al., 2008). In bears, increased levels of mir-27, mir29, and mir33 have also been recorded (Luu et al., 2020). These miR data provide a mechanism for a reversible gene silencing during torpor, rapidly reversed upon arousals, and for an active suppression of metabolism during the torpid state.

Reduction of enzyme activities due to posttranslational modifications like phosphorylation is a mechanism prone to lower the rate of ATP turnover during hibernation in small mammals (MacDonald and Storey, 1999; Lee et al., 2010). For instance, ion fluxes through membranes *via* ATPase pumps constitute a major energy-demanding process (Rolfe and Brown, 1997). In most tissues, including liver and muscle, the Na^+^/K^+^ and some calcium ATPases are downregulated in small mammals (Storey, 2010). Hibernation notably induces a 60% decrease of Na^+^/K^+^ ATPase activity in muscles of ground squirrels (*S. lateralis*), which appears regulated *via* reversible protein phosphorylation (MacDonald and Storey, 1999). Surprisingly, sarcoplasmic reticulum RyR1 and SERCA1 protein expression levels are higher, and major RyR1 and SERCA1 signaling pathway-related factors are active during torpor in Daurian ground squirrels (Wang et al., 2020) probably to handle the overload of calcium associated with complete inactivity and notably *via* periodic elevation in cytoplasmic calcium levels, which is normalized when individuals go back into torpor (see below). In black bear red blood cells, energy-saving mechanisms have been reported to involve decreased activities of Ca^2+^/Mg^2+^ ATPase and Na^+^/K^+^ ATPase (Chauhan et al., 2002). Possible inhibition of muscle Na^+^/K^+^ ATPase activity in hibernating brown bears (*U. arctos*) has been recently discussed, and abundance of the three isoforms of SERCA was shown to remain unchanged (Chazarin et al., 2019a). The possible modulation of SERCA activity by endogenous protein effectors like sarcolipin, by posttranslational modifications, or in relation with the lipid composition of the sarcoplasmic reticulum membrane having not been studied to date in hibernating bears.

Mitochondria is responsible for 90% of whole-animal oxygen consumption (Rolfe and Brown, 1997), therefore being an obvious target to study in relation with changes in MR (Staples and Brown, 2008; Staples, 2014). Mitochondria morphology is preserved in skeletal muscle of edible dormice during hibernation (Malatesta et al., 2009). However, mitochondrial respiration has been shown to drop during hibernation in small mammals (Staples, 2014), this suppression being 2- to 3-fold greater in intermyofibrillar than sub-sarcolemmal mitochondria in skeletal muscles from thirteen-lined ground squirrels (*Ictidomys tridecemlineatus*) (Brown and Staples, 2014). The investigation of mitochondrial respiration is still lacking for bears, however, the expression levels of nearly all mitochondrial respiratory chain complexes have been shown to drop in muscles from hibernating brown bears, in accordance with depressed metabolism (Chazarin et al., 2019a). Downregulation of muscle mitochondrial metabolism is also supported by a reduction of substrate oxidation during hibernation (Chazarin et al., 2019a). Hydrogen sulfide (H_2_S) is an endogenous gaseous molecule synthetized at the mitochondrial level, which has profound effects on mitochondrial respiration, either stimulatory at low dose or inhibitory at high dose (Murphy et al., 2019). By inhibiting the respiratory chain complex IV, exogenous H_2_S is able to induce a “hibernation-like” state in mice (Blackstone et al., 2005), reducing oxygen consumption without changes in T_b_ (Volpato et al., 2008). Stable total sulfide concentration in the μM range has been shown in the plasma from hibernating vs. active brown bears (Revsbech et al., 2014), thus suggesting H_2_S might help sustain a certain amount of mitochondrial electron chain activity during bear hibernation (Giroud et al., 2020). A role for respiratory acidosis in the inhibition of enzyme activities from the mitochondrial respiratory chain resulting in MR depression during hibernation has been hypothesized earlier (Malan, 1986, 2014), however, it has not been specifically studied in bears.

### Muscle Shivering and Protein Sparing During Hibernation

In humans, during bed rest of medium duration, physical exercise, and notably resistive vibration-induced small movements of muscles are able, when combined with whey/alkalizing salt supplementation, to prevent skeletal muscle protein changes, i.e., to limit muscle atrophy, and to maintain insulin sensitivity (Kenny et al., 2020). For small mammals, periodic arousals in deep hibernators, i.e., the return to euthermia and reactivation of major metabolic functions, and in particular the massive myofiber recruitment and elevated motor signaling during shivering in the early phase of arousals likely contribute to explain how, e.g., hibernating bats do not lose muscle mass relative to body mass during winter (Lee et al., 2010) (Figure 1). Although bears do not interrupt their fast and do not arouse during hibernation, it has already been hypothesized that the muscle preservation they exhibit may involve comparable mechanisms. It has already been mentioned above that muscle shivering occurs in hibernating bears (Harlow et al., 2004; Lin et al., 2004; Toien et al., 2011). Moreover, the nervous system exerts key control over skeletal muscles notably to maintain muscle tone and trigger muscle contraction, and the main consequence of muscle denervation is actually atrophy (Bongers et al., 2013). Bear skeletal muscles are sensitive to the atrophic effects of denervation during the summer-active period, however, not during hibernation (Lin et al., 2012). Therefore, the nervous system and, possibly, muscle shivering do not seem to be primarily involved in the resistance to muscle atrophy in hibernating bears.

### Urea Recycling and Protein Sparing During Hibernation

The limited decrease in muscle protein content may be at the origin of an elevation of 3-methylhistidine observed in the serum of hibernating vs. active brown bears (Hissa, 1997; Stenvinkel et al., 2013). However, unchanged or decreased levels of circulating urea, the main end product of protein catabolism, likely reflect low protein mobilization and recycling mechanisms for urea in bears during winter (Folk et al., 1976; Barboza et al., 1997; Hissa, 1997; Stenvinkel et al., 2013) (Figure 1). The coordinated underexpression of liver genes involved in the urea cycle during hibernation in black bears argues in favor of reduced urea production (Fedorov et al., 2009). Reduced expression of genes involved in urea production has also been recently reported in skeletal muscle from hibernating grizzly bears (*U. arctos horribilis*) (Jansen et al., 2019). In addition to decreased urea production due to limited protein catabolism, a rapid degradation of urea has been shown in hibernating bears (Wolfe et al., 1982; Stenvinkel et al., 2013), indicating the synthesis of essential amino acids, which can be used for gluconeogenesis (Nelson et al., 1975; Ahlquist et al., 1984). Finally, urea reabsorption, together with water and other substances, from the bladder epithelium to the bloodstream has been shown (Nelson et al., 1973, 1975). Many studies suggest that urea recycling also occurs in hibernating rodents (Galster and Morrison, 1975), urea cycle intermediates remaining at stable levels during torpor, which is consistent with the suppression of the urea cycle (Rice et al., 2020). Accordingly, genes involved in the urea cycle have been shown to be downregulated in the liver of torpid golden-mantled ground squirrel (Williams et al., 2005). The expression of urea cycle enzymes has also been shown to be less abundant and posttranslationally inactivated during hibernation in the liver of thirteen-lined ground squirrels (Epperson et al., 2010; Hindle et al., 2014), thus facilitating amino acid sparing during torpor. A potential for protein conservation through a urea recycling by the microbiome has been suggested in fasted and water-deprived golden-mantled ground squirrel (Riedesel and Steffen, 1980) (Figure 1).

Nitrogen recycling is suggested in hibernating bears by the greatly reduced amounts of urinary nitrogen and ammonia, which exceed changes in the blood (Nelson, 1973; Nelson et al., 1973; Folk et al., 1976) but also by normal concentration of blood total amino acids (Nelson et al., 1973; Hissa, 1997; Stenvinkel et al., 2013) and the absence of intestinal storage (Nelson et al., 1973). The nitrogen produced from amino acid and urea metabolism is likely used for amino acid synthesis and is incorporated in newly synthesized proteins (Lundberg et al., 1976; Nelson, 1978; Hellgren, 1998), a process likely involving glycerol released from mobilization of adipose fat stores (Ahlquist et al., 1984). In hibernating thirteen-lined squirrels, low levels of skeletal muscle AMP deaminase 1 (AMPD1), a protein regulating the AMP/ATP ratio, have been interpreted as reflecting an alteration in the production of nitrogenous waste (Anderson et al., 2016). Moreover, a selective enrichment of several essential amino acids in the plasma from hibernating ground squirrels has led the authors to hypothesize a mechanism whereby they are spared and recycled for use in new protein synthesis during the winter fast (Epperson et al., 2011). Nitrogen recycling has been investigated recently in hibernating arctic ground squirrels (Rice et al., 2020). It was notably shown that slow rates of skeletal muscle protein breakdown during torpor may provide a source of (essential) amino acids and that the free nitrogen released during protein breakdown is recycled and buffered by transamination, with accumulation in glutamine, glutamate, and alanine. After the infusion of [^15^N]ammonium acetate, the incorporation of nitrogen in amino acids and key energy metabolites during interbout arousals was in agreement with a *de novo* synthesis of amino acids and ultimately of proteins.

### Humoral Control of Muscle Protein Sparing During Hibernation

Together with metabolic hormones, a number of circulating factors are known to control muscle protein balance (Bonaldo and Sandri, 2013; Martin et al., 2018; Krause et al., 2019; Ibrahim et al., 2020b). They may therefore mediate protein sparing during hibernation (Figure 1). The serum concentration of testosterone is elevated during the second half of the hibernation period and then until mid-July, low values being thereafter recorded from mid-July to November–December (McMillin et al., 1976; Palmer et al., 1988; Garshelis and Hellgren, 1994; Tsubota et al., 1999). Similar results have been found in captive Hokkaido brown bears (*U. arctos yesoensis*) that are devoid of torpor, serum testosterone levels being gradually increased from February to April–May, then low baseline values being restored and remaining stable from the middle of the mating season (June) to January (Tsubota and Kanagawa, 1989). From these different studies, elevated levels of testosterone in the second half of hibernation period have been associated with testicular and spermatogenesis recrudescence in male bears. An increase in testosterone secretion also occurs in hibernating rodents before emergence (Barnes et al., 1988), which has also been associated with changes in spermatogenesis. Moreover, the increase in testosterone levels during hibernation is concomitant with an increase in skeletal muscle volume (Hindle et al., 2015). Testosterone, being known to promote protein synthesis (Dubois et al., 2012), has also been suggested to play a role in the control of blood urea and maintenance of the hibernating state in black bears (*U. americanus*) (Nelson et al., 1978).

In fat- and food-storing rodents, the secretion of most anabolic hormones, such as, e.g., insulin (Castex et al., 1984; Florant et al., 1986; Weitten et al., 2013) and insulin-like growth factor 1 (IGF1) (Schmidt and Kelley, 2001), is low during torpor. Insulinemia is also lower in hibernating bears compared to during the fall period of hyperphagia, while circulating glucagon levels tend to be higher during early hibernation (Palumbo et al., 1983; McGee-Lawrence et al., 2015). One study has found higher levels of circulating insulin in wild American black bears during hibernation (McCain et al., 2013), a characteristic that could be at the origin of the development of insulin resistance in bears (Rigano et al., 2017). Discrepant data concerning insulin concentrations may however be due to the absence of insulin assays specific to bear insulin. In addition, decreased expression of genes involved in muscle insulin signaling has also been recently shown in grizzly bears during hibernation (Jansen et al., 2019). Hence, the anabolic effects of insulin, in particular concerning protein synthesis, may therefore not be of key importance during bear hibernation. Decreased insulinemia may rather favor lipolysis during hibernation, as it is the case in response to fasting in non-hibernating rodents (Ibrahim et al., 2020a). Hibernating bears remain responsive to growth hormone (GH) treatment, but IGF1 serum levels decline in autumn to reach lowest values during early denning before they are increased in late denning to reach the highest values during summer (Donahue et al., 2006; Blumenthal et al., 2011; Seger et al., 2011). A role for the GH–IGF1 axis in fat accretion prior to denning and in the prevention of excessive lipolysis in early hibernation has thus been suggested in black bears. Because IGF1 is known to protect against muscle atrophy, notably *via* the control of intracellular protein balance (Timmer et al., 2018), such regulations may favor (muscle) protein sparing during bear hibernation.

Adiponectin is both an adipokine and myokine that is able to modulate metabolism and insulin sensitivity in skeletal muscle (Liu and Sweeney, 2014). Adiponectin has also been shown to protect skeletal muscles against atrophy, notably rodents *in vivo* and in C2C12 cells *in vitro* (Krause et al., 2019), a mechanism that does not seem to play a role in hibernating bears. Indeed, serum adiponectin levels have been shown to be related to body fat percentage in black bears, increasing gradually during the active season to reach highest values in the hyperphagic pre-denning period, then values were decreased back during hibernation (McGee-Lawrence et al., 2015; Rigano et al., 2017). Decreased adiponectinemia has also been shown in Scandinavian brown bears during hibernation vs. the summer-active season (Welinder et al., 2016). On the contrary, adiponectin is low at the beginning of fat accumulation and elevated during hibernation in fat- and food-storing rodents (Florant et al., 2004; Weitten et al., 2013), which could trigger fatty acid oxidation and inhibit gluconeogenesis during torpor (Fruebis et al., 2001; Masaki et al., 2003) and help to protect muscles.

Since total and free concentrations of thyroid hormones T3 and T4 have been shown to decline from October through March in the serum of American black bears, elevated levels being restored in April–May, it has been suggested that hibernating bears have a hypothalamic hypothyroidism (Azizi et al., 1979; Tomasi et al., 1998). Similar results have been found in Finnish brown bears (Hissa et al., 1994). Such a drop in thyroid hormone levels during denning is likely linked to depressed metabolism during hibernation. Because thyroid hormones are known to increase the number and diameter of muscle fibers (Salvatore et al., 2014) and muscle wasting is induced when they are deficient (Carneiro et al., 2008), it is difficult to understand if and how the regulation of the thyroid function during hibernation is involved or not in the control of bear muscle metabolism. Finally, osteocalcin, a bone-derived hormone, has been shown to promote muscle mass maintenance in aging mice, an effect likely due to stimulation of protein synthesis (Mera et al., 2016). Opposite results have been found in black and brown bears during hibernation, an increase in serum osteocalcin being observed for the former (McGee-Lawrence et al., 2015), whereas decreased osteocalcinemia was reported for the latter (Vestergaard et al., 2011). Osteocalcin might then play a role in muscle maintenance during hibernation in black bears, however not in brown bears. The reasons for such a difference between bear species are not understood.

Yet, the secret of muscle protein sparing in hibernating bears could be in proteolysis inhibition. The *ex vivo* incubation of rat skeletal muscle (extensor digitorum longus) for 120 min in a supplemented medium containing 5% of hibernating bear plasma has indeed resulted in the reduction of muscle net proteolytic rate (−40%) and of the levels of proteolytic-related mRNAs (cathepsin B, ubiquitin) as compared with rat muscles incubated with the plasma from non-hibernating bears (Fuster et al., 2007). The exposure of cultured human differentiated muscle cells to brown bear serum collected during winter and summer periods later confirmed that the anti-proteolytic properties of hibernating bear plasma/serum also affect human cells (Chanon et al., 2018). The protein turnover rate of human myotubes was reduced when they were incubated during 48 h with the serum from hibernating vs. summer-active bears, a dramatic inhibition of proteolysis involving both proteasomal and lysosomal systems being observed, which resulted in an increase in muscle cell protein content. Therefore, the plasma from hibernating bears appears to contain one or several proteolytic inhibitors that may modulate muscle intracellular pathways to attenuate muscle loss during hibernation. Cortisol (or corticosterone) is the main steroid hormone that is involved in protein degradation (Schiaffino et al., 2013). In fat-storing rodents, plasma concentrations of cortisol are low during hibernation compared to the active season, which might help prevent protein catabolism (Shivatcheva et al., 1988; Armitage, 1991). In bears, however, values of corticosteroids were observed to be higher during fall hyperphagia and hibernation periods than during the summer-active phase (Palumbo et al., 1983; Harlow et al., 1990; Hellgren et al., 1993; Donahue et al., 2003a,b), which has been proposed to be involved in lipolysis promotion, rather than gluconeogenesis from the use of amino acids (Harlow et al., 1990). Other anti-proteolytic factors remain therefore to be found.

### Intracellular Pathways of Muscle Protein Sparing During Hibernation

Several intracellular signaling pathways that could be involved in the regulation of muscle protein balance in hibernators have been listed elsewhere (Tessier and Storey, 2016; Gonzalez-Bernardo et al., 2020). In bears, the elevated expression of multiple genes involved in protein biosynthesis and ribosome biogenesis has been consistently documented in skeletal muscles from hibernating black bears (Fedorov et al., 2009, 2014) and grizzly bears (Jansen et al., 2019). Moreover, an increased level for the phosphorylated form of S6K1 was observed in the skeletal muscles of hibernating Japanese black bears (*Ursus thibetanus japonicus*) (Miyazaki et al., 2019), suggesting an activation of mammalian target of rapamycin (mTOR) complex 1 (mTORC1) signaling. Accordingly, the gene expression of myostatin, a negative regulator of skeletal muscle growth that reduces mTORC1 activity *via* Smad2/3 signaling (Trendelenburg et al., 2009; Welle, 2009), was significantly downregulated following hibernation. In thirteen-lined ground squirrels (*Spermophilus tridecemlineatus*), no significant change of myostatin expression was observed during entrance into torpor and early or late torpor (Brooks et al., 2011). There was, however, approximately a 6-fold increase in myostatin protein levels as squirrels arose from torpor. If the absence of variation during torpor goes in the direction of muscle preservation, the increase during arousal phases does not support the hypothesis that shivering thermogenesis promotes muscle anabolic pathways (Lee et al., 2010). Mechanisms that could release the suppression or promote increased levels of myostatin involved the upregulation of both Smad2 and phosphorylated Smad2 during early torpor, but only that of phosphorylated Smad2 during arousals (Brooks et al., 2011). Overall, these regulations likely enhance muscle growth following hibernation and counteract an excessive loss of muscle during hibernation. Transcriptional changes affecting genes associated with insulin–Akt–mTOR signaling in skeletal muscle of hibernating grizzly bear and changes in non-essential amino acid levels have recently been suggested as cooperating mechanisms that may explain the increase in mTOR activity during hibernation (Mugahid et al., 2019). Whereas unchanged transcriptional level of protein catabolism genes was recorded in black bears (Fedorov et al., 2009, 2014), increased mRNA levels have been reported for key factors of the ubiquitin-proteasome and autophagy degradation pathways in sartorius muscle of Japanese black bears at the end of the hibernation period, which may contribute to the decrease in mass of this muscle (see above) (Miyazaki et al., 2019). In grizzly bears, skeletal muscle mRNA levels have been observed decreased for muscle protein degradation pathways (Jansen et al., 2019). A coordinated downregulation of atrogenes during arousal has also been reported in arctic ground squirrels (Goropashnaya et al., 2020). Overall, these data suggest that, if an inhibition of proteolysis is involved in muscle protein sparing during hibernation (see above), part of the regulation is made at the transcriptional level for brown bears and arctic ground squirrels, albeit not for black bears.

In small mammal hibernators, a previous study examined some parameters of transcriptional control in skeletal muscle of thirteen-lined ground squirrels that could be used to provide reversible suppression of transcription during torpor (Morin and Storey, 2006). The authors notably reported a significant reduction of maximal activity of RNA polymerase II in muscles during hibernation to just 58% of the activity in euthermic muscle despite a constant total amount of Pol II protein. Moreover, RNA content is decreased during small mammal hibernation, suggesting a loss of functional ribosomes as observed in suspended rats (Steffen and Musacchia, 1984), impairing protein synthesis. Recent studies show, however, that this seems to be dependent on the species and muscle studied, and that it evolves during hibernation. Indeed, a recent study did not support an arrest of transcription during low-temperature torpor in quadriceps muscle of hibernating arctic ground squirrels but supported a transcriptional elevation of protein biosynthesis (Goropashnaya et al., 2020). The reduction of the levels of AMPD1 preceding and during hibernation in thirteen-lined squirrels, by reducing the ability to clear cellular AMP, could lead to increased AMPK activity and could thereafter induce a reduction of muscle mass *via* an inhibition of protein synthesis (Anderson et al., 2016). This said, if muscle protein synthesis decreases at the start of hibernation, it then increases again during late hibernation through the activation of the mTOR signaling cascade, as shown in hibernating bats and rodents (Lee et al., 2010; Nowell et al., 2011; Fedorov et al., 2014), leading to an increase in skeletal muscle volume before emergence (Hindle et al., 2015). A role for fibromodulin in the muscle maintenance seen during hibernation has also been hypothesized in thirteen-lined squirrels (Anderson et al., 2016). The activation of serum/glucocorticoid-induced kinase 1 (SGK1), an activator of mTOR signaling, has also been shown in hibernating thirteen-lined ground squirrels (Andres-Mateos et al., 2013). Moreover, the phosphorylation levels of Akt1 (*p*-Akt1) and mTOR (*p*-mTOR) are reduced significantly in bats during hibernation compared to summer-active bats (Lee et al., 2010). Upon arousals, little variations of *p*-Akt1 occur, but *p*-mTOR exhibits biphasic oscillations. These findings suggest that the resistance to atrophy in small deep hibernators might be attained primarily by relatively constant or decreased proteolysis (see below) in combination with oscillatory anabolic activity (e.g., *p*-mTOR) during arousals. This response is not restricted to skeletal muscle, as both cardiac (Wu and Storey, 2012) and smooth muscle (Talaei, 2014) also show increases in mTOR activity upon arousal from torpor. Muscle preservation during torpor in thirteen-lined ground squirrels may also involve an increase in the percentage of satellite cells favoring growth processes (Brooks et al., 2015). Finally, an activation of the endurance exercise pathway involving PGC-1α has been shown in hibernating 13-lined ground squirrels (Xu et al., 2013), and this might therefore be an important mechanism for the preservation of skeletal muscle during hibernation.

Protein degradation has been reported either to decrease during small mammal hibernation, thus contributing to protein sparing, or to remain unaltered. Indeed, the activity of the ubiquitin-proteasome system is largely suppressed at low T_b_ during small mammal torpor (Velickovska et al., 2005), which is expected to greatly reduce muscle catabolism during torpor bouts. Muscle calpain activity also appears inhibited possibly due to elevated levels of its specific inhibitor, calpastatin, in hibernating Daurian ground squirrels (Yang et al., 2014). Moreover, the skeletal muscles of hibernating Daurian ground squirrels maintain protein sialylation homeostasis by restoring sialylation modification upon periodic arousals, which might help prevent disuse atrophy (Zhou et al., 1997). A decrease in protein degradation can be attributed to the downregulation of the FOXO1 proteolytic pathway (Nowell et al., 2011). In this context, the levels of proteolysis-related factors in skeletal muscles, including phosphorylated FOXO1 (*p*-FOXO1), atrogin-1, MuRF1, Skp2, and calpain-1, seem to remain constant during hibernation in bats (Lee et al., 2010). In addition, SGK1 activation in hibernating thirteen-lined ground squirrels has been involved in downregulation of proteolysis and autophagy (Andres-Mateos et al., 2013). In the Daurian ground squirrel during hibernation, the expression of IKKβ and MuRF1 remains stable in little atrophied soleus and extensor digitorum longus muscles, whereas an increase in MuRF1 mRNA level in the soleus and MuRF1 protein level in the extensor digitorum longus is observed upon arousals (Wei et al., 2018). Stress conditions experienced by animals can trigger excessive endoplasmic reticulum stress, which can lead to the activation of the unfolded protein response to adapt cellular folding capacity, but it can also drive cell death (Karagoz et al., 2019). In this regard, the upregulation of *p*-eIF2α and GRP78 mediated by PERK signaling pathway has been identified in torpid Daurian ground squirrels as possibly alleviating elevated endoplasmic reticulum stress, hence preventing skeletal muscle cell apoptosis during hibernation (Zhang et al., 2019b). There was also an indication for an inhibitory role of apoptosis during the torpid state, as suggested by a reduced Bax/Bcl-2 protein ratio in muscles of torpid Daurian ground squirrels, which was recovered upon interbout arousals (Fu et al., 2016).

Intracellular calcium overload plays an important role in the mechanisms of disuse-induced muscle atrophy. Increased intracellular calcium concentration can activate some proteases (e.g., calpains) and trigger the catabolism of myofibrillar proteins (Costelli et al., 2005; Goll et al., 2008; Fu et al., 2016). Prolonged skeletal muscle disuse (e.g., during spaceflight, hind limb unloading, and bed rest) can notably lead to disturbance of intracellular calcium homeostasis, mainly exhibited by cytoplasmic calcium overload (Goll et al., 2003; Costelli et al., 2005; Shenkman and Nemirovskaya, 2008). Interestingly, hibernators might regulate intracellular calcium homeostasis during hibernation *via* transient cytosolic overload of calcium upon arousals. For instance, a previous study in Daurian ground squirrels showed increased cytoplasmic calcium levels in skeletal muscle fibers during late torpor and upon interbout arousals and partial recovery when the animals entered torpor again (Zhang et al., 2019a; Wang et al., 2020).

The resistance to muscle atrophy in hibernating bears has also been shown to involve other mechanisms. The expression levels of pyruvate dehydrogenase kinase 4, a protein known to inhibit the activity of pyruvate dehydrogenase, thereby limiting the entry of glycolytic intermediates into the Krebs cycle, is increased in skeletal muscles from hibernating Japanese black bears (Shimozuru et al., 2016) and hibernating Scandinavian and American brown bears (Chazarin et al., 2019a; Mugahid et al., 2019). These data, together with the increase in muscle lactate dehydrogenase activity levels and either maintained or reduced plasma lactate levels (Ahlquist et al., 1984; Evans et al., 2012; Chazarin et al., 2019a), have led some authors to suggest that the Cori cycle is active in bears during hibernation, therefore helping to spare muscle protein during hibernation (Shimozuru et al., 2016; Chazarin et al., 2019a). Previous data indicated an enhancement, *via* a COX-2-dependent pathway, of *in vitro* skeletal muscle growth in C2C12 myotubes where the supplementation with arachidonic acid triggers increased secretion of prostaglandin F2a and prostaglandin E2 (Markworth and Cameron-Smith, 2013). Accordingly, the maintenance of prostaglandin levels in skeletal muscles from hibernating brown bears has been proposed to play a role in muscle sparing (Giroud et al., 2018). Moreover, a role for increased levels of omega-3 fatty acids like docosahexaenoic acid (DHA) and docosapentaenoic acid has been suggested recently in hibernating brown bears (Chazarin et al., 2019a). DHA-enriched diets are known to help preserve muscle integrity in various catabolic conditions (Smith et al., 2015; Deval et al., 2016) and to induce muscle protein synthesis (Wei et al., 2013). DHA itself also prevents palmitate-induced proteolysis *in vitro* (Woodworth-Hobbs et al., 2017). Furthermore, hibernation has been shown to elicit a myogenic microRNA, or “myomiR,” response *via* MEF2A-mediated signaling in skeletal muscle from hibernating brown bears, consisting of an upregulation of miR-1 and miR-206 and respective downregulation of pax7 and id2 mRNAs (Luu et al., 2020). Increased levels of miR-206, miR-221, and miR-31 were also indicative of stimulated muscle regeneration process, and higher levels of miR-33, miR-23a, and miR-29b suggested suppression of ubiquitin ligase expression. A significant role for MEF2-mediated gene transcription has also been suggested in hibernating ground squirrels (Tessier and Storey, 2010). MEF2 protein levels significantly increased when animals were in torpor and the amount of phosphorylated (Thr312) active MEF2A increased during entrance into torpor. MEF2C levels also rose significantly during entrance into torpor and torpor as did the amount of phosphorylated MEF2C Ser387.

Finally, because oxidative stress promotes muscle proteolysis and inhibits protein synthesis in catabolic conditions (Powers et al., 2016), its limitation in skeletal muscle from hibernating brown bears has been proposed to favor resistance to atrophy (Chazarin et al., 2019b). More precisely, increased gene expression for the cold-inducible proteins CIRBP and RBM3 was observed and could favor muscle mass maintenance and alleviate oxidative stress during hibernation. In addition, a possible reduction of the production of reactive oxygen species in the hibernating muscle could be hypothesized from indices of reduced mitochondrial content. It was paralleled by a coordinated induction of cytosolic antioxidant systems, under the control of the transcription factor NRF2, and the maintenance of the GSH/GSSG ratio. H_2_S is known to have antioxidant properties (Murphy et al., 2019). It has been suggested previously that remodeling of H_2_S metabolism could be involved in the synthesis of GSH found in high quantity in hibernating bear erythrocytes (Revsbech et al., 2014). In bear muscles, the GSH/GSSG ratio was maintained stable during hibernation due to decreased levels of both glutathione forms (Chazarin et al., 2019b), and an eventual role for H_2_S therefore remains undetermined. Lower levels of oxidative damage were recorded in hibernating bear skeletal muscles (Chazarin et al., 2019b). Enhanced expression of chaperones and heat shock proteins in bear skeletal muscle during hibernation may also help maintain the integrity of muscle proteins (Chazarin et al., 2019b). It is noteworthy to mention that antioxidant-rich berries (Rimando et al., 2004; Zifkin et al., 2012) are consumed in large amounts by bears during the summer and autumn (Stenset et al., 2016). Whether and to what extent such a diet prior to denning has an influence on bear muscle protection against oxidative stress during hibernation are not yet known. Antioxidant mechanisms have also been hypothesized in small hibernating mammals, involving, for example, allantoin and ascorbate (Epperson et al., 2011).

Altogether, along the hibernation period in small as well as in large hibernators, these molecular and subcellular mechanisms contribute to the sparing of muscles *via* tight controls of both the synthesis and the catabolism of proteins (Figure 1).

## Hibernators as Good Models to Study the Maintenance of Muscle Mass

Bernard (1865) and Krogh (1929) were among the first, if not the first, to develop the idea that the solution to solve a problem in biology or medicine is necessarily hidden in an animal species, hence pioneering the biomimicry concept. Hundred years later, and particularly since the discovery that mole rats have anticancer mechanisms that do not exist in mice or rats, i.e., the so-called standard models, there is an increasing interest for “exotic” animals as a source of biomedical innovation (Sedivy, 2009). In this context, hibernation is a unique metabolic condition, since it is superimposing an important drop in both energy expenditure and physical activity to a usually long fast. From the pioneering work of Cahill (1970), it is well-known that the main adaptation to tolerate a long fast in humans is a decrease in protein breakdown, the so-called “protein sparing” through the mobilization of body fat. But, in contrast, inactivity results in an important reduction in muscles (Rudrappa et al., 2016).

### Protein Sparing and Fasting Abilities in Hibernators

The first remarkable feature of hibernators is, as indicated above, that they are able to rely essentially on their body fat despite inactivity. They consistently emerge from winter with very little atrophy despite conditions that would typically result in muscle atrophy combined with a loss of oxidative fibers (Cotton, 2016). A major question was whether the extent of the drop in T_b_, i.e., torpor vs. deep hibernation, interferes with this protein sparing. Quantitative data are limited to answer this question, particularly in small hibernators, due to the difficulty in monitoring small changes in body condition. Importantly, therefore, in shallow- and deep-hypothermal fasting hedgehogs (Cherel et al., 1995), despite the different levels in energy expenditure of about 2.5 and 1 W.kg^−1^, respectively, the energy sources were found identical in both groups, neutral lipid contributing similarly as the main fuel (about 90%) and body protein accounting for the same remainder (about 10%). In the fat-storing arctic ground squirrel, the contribution of body protein to energy expenditure during hibernation is also about 10% (Galster and Morrison, 1976). It therefore suggests that the extent of the drop of energy expenditure does not interfere with the efficiency in protein sparing.

**Figure 2:**
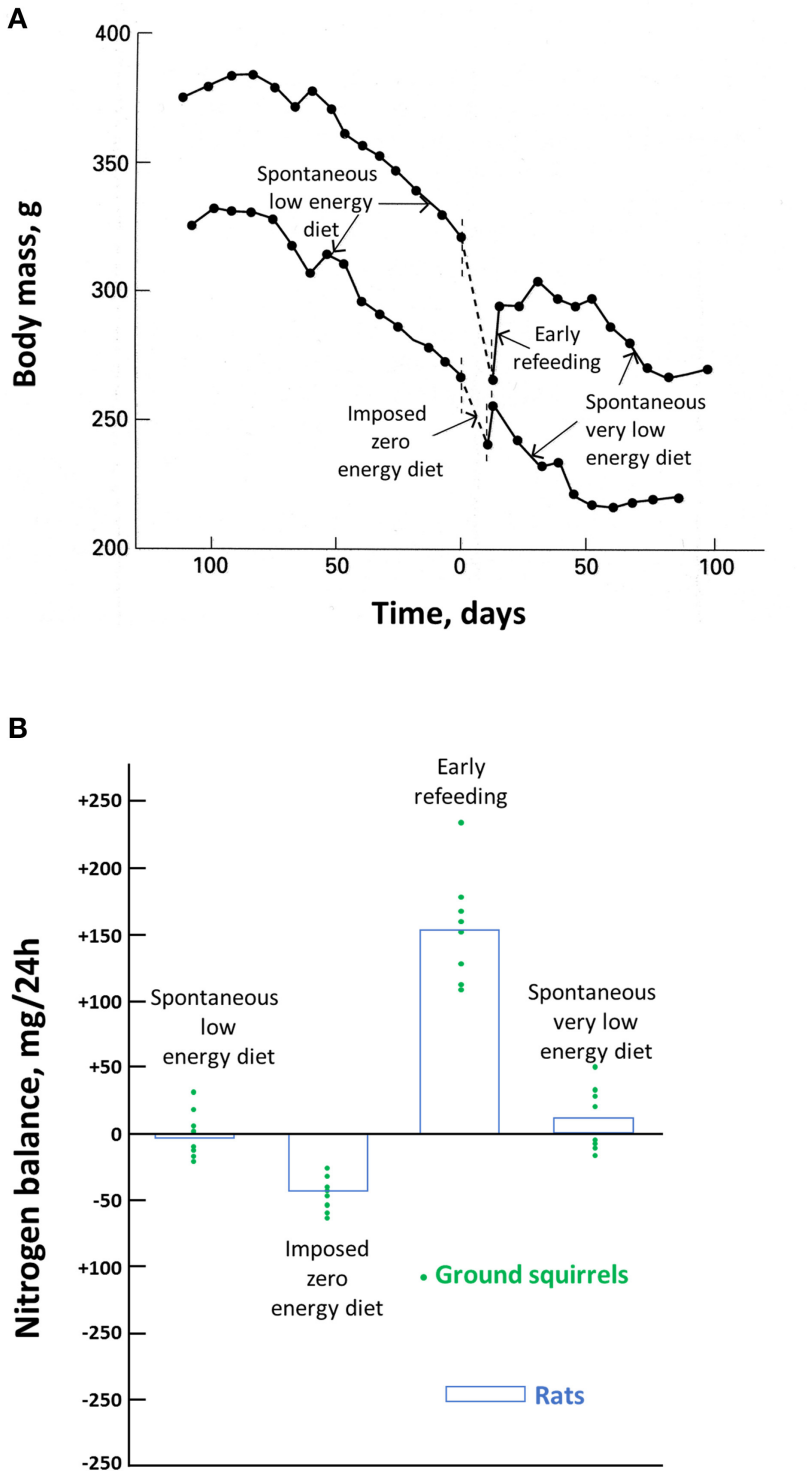
Effects of food deprivation in ground squirrels and rats. Body mass changes in two hibernating ground squirrels before, during, and after an experimental fast are shown in **(A)** and the nitrogen balance in hibernating ground squirrels before, during, and after an experimental fast, and in experimentally fasted rats are shown in **(B)**. Modified from Karmann et al. (1994).

The maintenance of body protein, and particularly muscle mass, is critical, as human death occurs when about 50% of body proteins have been depleted (Silber, 1984). As for hibernating species during torpor, obese and non-obese humans are very efficient in their ability to spare body proteins during food deprivation, and they may safely undergo therapeutic fasts of 1 to 3 weeks (Wilhelmi de Toledo et al., 2019). However, due to the high caloric value of fat compared to body protein, severely obese humans would not be able to undergo such a fast as long as necessary to deplete their excessive fat. Long before, they would have reached this critical stage where about 50% of body protein have disappeared and die (Le Maho et al., 1988). One way for severely obese humans to rapidly lose fat in excess while reducing protein loss is therefore by undertaking a very low energy diet (VLED) providing protein. The ambition is not to totally preserve body protein but to minimize its loss, and it is more efficient with a behavioral program that involves increased locomotor activity (Asher et al., 2013; Parretti et al., 2016). In this biomedical context, it is therefore of particular interest that food-storing hibernators, such as golden-mantled ground squirrels (*Citellus lateralis*), which can survive for months without eating, have a very low food intake during their episodes of arousals (Mrosovsky and Sherry, 1980; Kauffman et al., 2004). This spontaneous drop in food intake is not specific of hibernators, since it also occurs in non-hibernator animals, such as, for birds, the female of the red junglefowl (*Gallus gallus*) when incubating its eggs (Mrosovsky and Sherry, 1980). It is a spontaneous fast, i.e., an anorexia, since the squirrel and the bird do not eat more when food is nearby *ad libitum*. In both cases, food intake is progressively decreasing from a figure, which corresponds to a low energy diet (LED) to a value that can be later considered as a spontaneous VLED, since food intake is reduced by 80% or more. Consequently, body mass is decreasing during the entire period when torpor occurs in squirrels and all throughout the incubation for the junglefowl. If food is removed, which therefore corresponds to a zero energy diet, there is obviously an accelerated drop in body mass (Figure 2A) (Karmann et al., 1994). When food is again available, food intake is temporarily larger than it was before. It is however quite remarkable that food intake returns to the previous spontaneous very low caloric value once body mass has reached not its value at the onset of the zero-calorie diet but the lower value it would have reached at that time without any interruption of the VLED. This means therefore that there is a sliding set point in the defense in body mass during spontaneous VLED.

Considering the physiological mechanisms involved in this regulated drop in body mass, it was therefore interesting to determine the metabolic consequences of the spontaneous VLED. Indeed, in obese humans, the aim of VLEDs is to minimize body protein depletion in association with a reduced calorie intake in order to reduce excessive fat. Again, as indicated above, although fat contributes to most of the energy expenditure in obese humans under a zero-calorie diet, the low caloric value of body protein would result in a critical loss in lean body mass while fat is still in excess (Le Maho et al., 1988). Moreover, as documented in this review, hibernators, as birds before breeding or migration, generally store a huge amount of fat. Metabolic flexibility appears therefore as a prerequisite for body protein savings during hibernation.

The most accurate way to determine individual changes in body protein is through a nitrogen balance, i.e., a comparison of nitrogen intake and output. This has been done during the season of torpor in ground squirrels (Figure 2B) (Karmann et al., 1994). It shows that all through that period, the spontaneous LED and then VLED that occur during each arousal compensate for the loss in body protein during the previous episodes of torpor. On a zero-energy diet, there is obviously a significant loss in body protein, although much smaller than that in rats, which, in contrast to the squirrels, are not hibernators. There is an initial high food intake once food is again available. It allows to restore this larger than usual body protein loss. But the return of body mass to the defended value corresponds again to the situation when the spontaneous VLED allows to compensate for the protein loss during torpor. It accords with the observation that dry fat-free body mass is maintained in those hibernators hoarding food, which allows them to feed during arousals (Jameson and Mead, 1964). It is unfortunate that, to our knowledge, there are no more investigations on the nitrogen balance of food-storing hibernators. Based on the similar contribution of body protein and fat in torpid and hibernating hedgehogs (Cherel et al., 1995), we might however assume that fat-storing deep hibernators manage as well as the squirrels after torpor episodes to compensate from the protein loss that has occurred during deep hibernation and arousal. In the absence of nutrient intake, provision of amino acids and prevention of ammonia toxicity in hibernating arctic ground squirrels have recently been shown to involve buffering and recycling of free nitrogen released from skeletal muscle protein breakdown during torpor into newly synthesized amino acids and proteins during interbout arousals (Rice et al., 2020).

Finally, by relying on a reduced energy diet during the episodes of arousal, food-storing hibernators seem to be able to achieve the same goal, as do the hibernating bears in maintaining body protein and only relying on fat without eating. Elucidating how small and larger hibernators manage to be so efficient is therefore an important biomedical goal.

### Transfer of Hibernator Protein-Sparing Abilities to Humans

**Figure 3:**
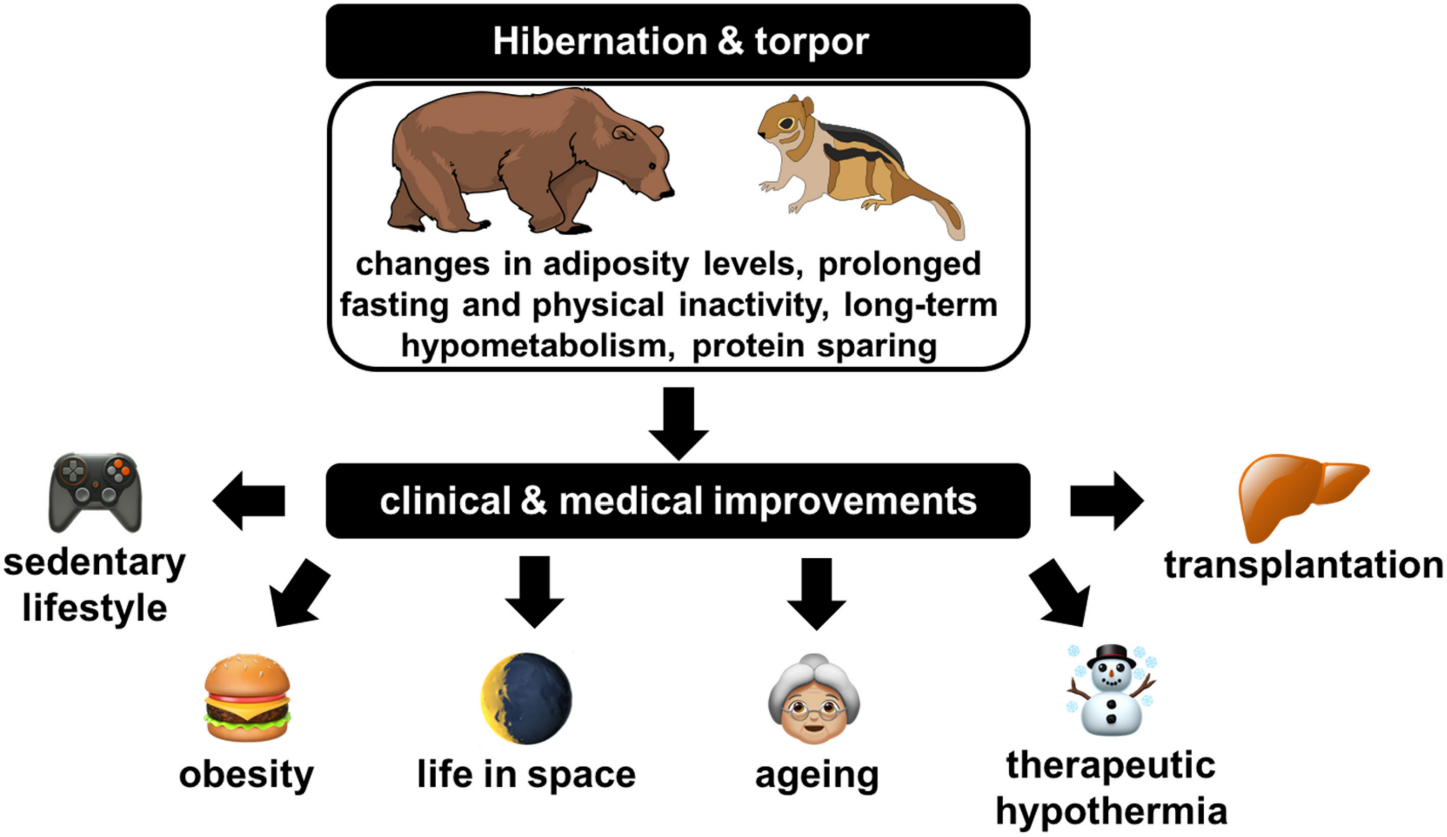
Illustration of the clinical and medical improvements that could likely originate from a deep understanding of physiological, cellular, and molecular regulations at play in hibernators.

Apart from the deprivation of food, innovations inspired from the study of hibernation can be expected to improve a number of other situations where (muscle) proteins are lost in humans, including, e.g., during aging, sedentary lifestyle, and life in space and for the preservation of tissues and organs for transplantation (Figure 3). In the context of translational research, some of the aspects and benefits of considering hibernators as models of choice to better understanding and managing diseases (e.g., ischemia–reperfusion injury, chronic kidney disease, cardiovascular diseases, obesity/diabetes) or pathophysiological situations (e.g., muscle disuse) in humans have been discussed elsewhere (Carey et al., 2003a; Bodine, 2013; von Linde et al., 2015; Stenvinkel et al., 2018).

Millions of people suffer from muscle atrophy worldwide (Ding et al., 2018), and this number is expected to increase rapidly. Apart from the widespread chronic diseases like cardiovascular diseases or cancers, the main causes of muscle loss remain aging, malnutrition (in the form of undernourishment or overeating), and physical inactivity, the prevalence of which is continuously growing. First, there is a general trend toward population aging throughout the world, with the number of persons aged 80 years or over being projected to increase more than 3-fold between 2017 and 2050, rising from 137 to 425 million (United Nations, 2017). Second, almost 690 million people in the world (~9% of the world population) were estimated to have been undernourished in 2019, and projected values indicate that more than 840 million people could be undernourished in 2030 (FAO et al., 2020). Third, global obesity has more than doubled since the 1980s, with more than 1.5 billion overweight adults, and current estimates are not optimistic as they still predict an increase in the prevalence of obesity of 33 and 130% for severe obesity by 2030 (Finkelstein et al., 2012). Fourth, sedentary lifestyles and the lack of physical activity have become a threat to public health, reaching pandemic proportions, making it now considered the fourth leading cause of morbidity and mortality in the world (Kohl et al., 2012). Weightlessness is also a well-known cause for skeletal muscle atrophy (Narici and de Boer, 2011), which for now remains among the limiting factors in the context of the human exploratory missions to the moon or Mars, which are today envisioned by almost all major national and international space agencies in the world as well as private companies (Patel et al., 2020).

The high prevalence of muscle wasting and its consequences such as impairment of health and life quality, the induction of weakness, fatigability, frailty, reduced activity, and metabolic alterations, and delayed recovery from diseases have led to decades of research dedicated to a better understanding of associated mechanisms (Powers et al., 2016; Ji and Yeo, 2019; Vainshtein and Sandri, 2020). Physical inactivity has been reported as a primary cause for developing metabolic inflexibility during bed-rest studies (Bergouignan et al., 2011), disuse-induced muscle atrophy being associated with the development of insulin resistance, hyperlipidemia, and a decreased capacity for oxidizing lipids (Stein and Wade, 2005). Insulin resistance and impairment in muscle lipid oxidation are also well-known features of obesity (Houmard, 2008). Although lean body mass is generally higher in overweight or obese individuals compared to their lean counterparts (Forbes and Welle, 1983), muscle quality and function are generally deteriorated (Cava et al., 2017). Moreover, obesity has been shown to progressively induce muscle atrophy, notably due to decreased physical activity, muscle inflammation, and impairment of muscle protein synthesis (Kim et al., 2014, 2016; Roy et al., 2016; Cava et al., 2017). Defining sarcopenia as the loss of muscle mass and strength and, ultimately, function, muscle wasting associated with obesity has led to the notion of sarcopenic obesity (Baumgartner, 2000). In obese individuals, sarcopenia is notably exacerbated by lipotoxicity to muscle cells due to increased fat levels (Prado et al., 2012). Sarcopenic obesity adverse effects are multiple, with notably increased risks of developing insulin resistance, metabolic syndrome, dyslipidemia, inflammation, and cardiovascular diseases in comparison with obesity or sarcopenia alone (Roh and Choi, 2020). Given the actual pandemic of obesity and its associated comorbidities, it is of crucial interest to develop efficient therapies and treatments for ensuring human health. Weight loss therapy is generally at the forefront to resolve obesity. However, body weight loss is usually achieved *via* a reduction in both fat mass and lean body mass (Cava et al., 2017; Willoughby et al., 2018). A substantial loss of lean body mass can negatively affect overall metabolism, muscle function, and other physiological processes, and it may worsen body fat accretion (Cava et al., 2017; Willoughby et al., 2018). The use of food deprivation to treat obesity has proven successful in some cases (Stewart and Fleming, 1973); however, the occurrence of several sudden deaths has been recorded (Cubberley et al., 1965). As with anorexia nervosa (Silber, 1984), the cause of these deaths has been attributed to excessive loss of body proteins (Le Maho et al., 1988). Therefore, adequate therapies should trigger fat mass loss in a sustainable way while preserving muscle mass and strength. As already stressed in this review, the continuously growing basic knowledge of the cellular and molecular mechanisms of muscle atrophy (Bonaldo and Sandri, 2013) and a myriad of wasting conditions including, e.g., prolonged fasting (Ibrahim et al., 2020b), physical inactivity (Rudrappa et al., 2016), and microgravity (Tascher et al., 2017) could be a way to identify possible anti-atrophy targets. From such studies, clinical therapies to fight muscle atrophy have been developed, notably including physical exercise, nutritional supplements, and a series of drugs (Carraro et al., 2018; Cretoiu and Zugravu, 2018; Kern et al., 2018; Owens, 2018; Rabelo et al., 2018; Sakuma and Yamaguchi, 2018; Shen et al., 2018). Nonetheless, no effective treatments have been proven to fully prevent loss of muscle mass (Ding et al., 2018; Duan et al., 2020).

On the reverse, hibernators are naturally resistant to large changes in adiposity levels (obesity is a necessary part of life for most hibernating animals) and to skeletal muscle atrophy despite long periods of dropped MR accompanied by fasting and physical inactivity, abilities that likely have an evolutionary origin. We should therefore get inspired from the “turning on” of specific regulatory programs involved in the control of energy and protein metabolism in hibernating animals (see above) and the “turning off” of other programs involved in the development of obesity comorbidities (Figure 3). Hence, molecular studies in hibernating mammals have already been proven successful in identifying potential therapeutic targets for combatting loss of skeletal muscle mass associated with muscle degeneration and atrophy. It has been demonstrated that SGK1, which appears involved in muscle preservation of hibernating 13-lined ground squirrels, is also critical for the maintenance of skeletal muscle homeostasis and function in non-hibernating mammals in normal and atrophic conditions such as starvation and immobilization (Andres-Mateos et al., 2013). Research in hibernating mammals will probably bring a wealth of new knowledge in the future, and it may result 1 day to the application of critical hibernator strategies to human wasting conditions. Therapeutic hypothermia has already been designed in various clinical contexts (Turban et al., 2001; Lee and Ding, 2020). Going further, i.e., by mimicking a hibernation-like state or inducing relevant hibernation features in humans could be of great help to favor healthy aging, improve health outcomes in critical patients, long-term immobilized patients, sedentary and obese people, and to promote healthy spaceflight to deep space (Martin, 2008; Johnson et al., 2013; Wu et al., 2013; Gorr, 2017; Chouker et al., 2019; Ferris and Gregg, 2019; Al-attar and Storey, 2020) (Figure 3).

Proteolysis is one of the main limitations for the preservation of allografts (Calmus et al., 1995), and suppression of major metabolic functions is a goal to reach to allow extending preservation times (Guibert et al., 2011). Apart from their natural resistance to muscle atrophy, hibernators are also able to preserve the functionality of their organs over the long-term while into a hypometabolic state notably through body protein sparing *via* inhibition of protein degradation (see above). They therefore constitute very good models for the improvement of the preservation of tissues and organs for transplantation (Ratigan and McKay, 2016; Soo et al., 2020) (Figure 3). If interbout arousals during hibernation in small mammals have a lot to teach regarding notably ischemia–reperfusion injury problems in the context of the cold storage of organs (Zancanaro et al., 1999; Soo et al., 2020), the concept of normothermic or sub-normothermic organ preservation has gained much attention in recent years. Not only normothermic organ conservation overcomes the main weaknesses of cold storage, e.g., by avoiding ischemia–reperfusion injury (Vogel et al., 2012), but it also allows much longer preservation times. It therefore appears as a very promising way to overcome the current shortage of organ donors worldwide while enabling to provide transplantation for a greatly increased number of patients (Brockmann et al., 2009; Nasralla et al., 2018). As they hibernate with a slight T_b_ lowering, bears and lemurs appear as very good models to help improve the field of warm (sub-normothermic to normothermic) organ preservation (Hadj-Moussa and Storey, 2019). With a core T_b_ greater than 30°C during hibernation, bears are indeed not confronted with the need for periodic body rewarming to revert deep hypothermia and associated adverse effects of torpor such as in small hibernators (see above). Neither they arouse nor return to euthermia after entering torpor, but they have been proven to be successful in preserving organ functionality over several months while into the torpor state.

## Conclusion

Based on the description above, it clearly appears that hibernators constitute unique animal models to study energy and substrate metabolism associated with muscle preservation under extreme conditions of complete inactivity and/or food deprivation. They provide avenues for further investigations with obvious biomedical interests, including treatments for obesity and cardiovascular diseases, and for improving health conditions during space travels. Bears seem to be unique models to develop new ways of organ preservation and to study the preservation of skeletal muscle during complete inactivity at mild T_b_. Some hibernating primate species, as, e.g., lemurs from the *Cheirogaleus* family, including fat-tailed dwarf lemur (*Cheirogaleus medius*) and greater dwarf lemur (*Cheirogaleus major*), may also represent good models to that respect due to their phylogenetic proximity with humans. Small hibernating rodents appear as useful models to discover new ways to improve therapeutic hypothermia due to very low T_b_ during torpor. As they repeatedly experience periodic arousals that seem to interfere with protein metabolism, their study is also expected to improve the understanding of metabolic depression and muscle preservation. The small size of deep hibernators may also represent an advantage due to a usually easy handling compared to large hibernators or other heterothermic primates.

The underlying mechanisms of muscle preservation at the molecular/cellular level and at the more integrative level during the torpor state appear to slightly differ, if not substantially, between the two types of hibernators. Muscle preservation involves mechanisms of protein sparing that are specific to hibernating bears and a combination of reduced energy expenditure and protein synthesis-related compensatory processes in small hibernators. Therefore, the development of comparative studies investigating these mechanisms in a variety of heterotherms (present across a large spectrum of species from mammals, birds, and marsupials) and considering the diverse forms of hibernation or torpor known to date would largely contribute to elucidating the extraordinary ability of some hibernators to spare protein and conserve their muscles during hibernation.

## Author Contributions

All authors listed have made a substantial, direct and intellectual contribution to the work, and approved it for publication.

## Conflict of Interest

The authors declare that the research was conducted in the absence of any commercial or financial relationships that could be construed as a potential conflict of interest.

## Abbreviations

AMPD1: AMP deaminase 1
BAT: brown adipose tissue
CO_2_: carbon dioxide
DHA: docosahexaenoic acid
GH: growth hormone
H_2_S: hydrogen sulfide
IGF1: insulin-like growth factor 1
LED: low energy diet
MR: metabolic rate
mTOR: mammalian target of rapamycin
mTORC1: mammalian target of rapamycin complex 1
*p*-Akt1: phosphorylated AKT1
*p*-FOXO1: phosphorylated FOXO1
*p*-mTOR: phosphorylated mTOR
RQ: respiratory quotient
SERCA: sarcoplasmic/endoplasmic reticulum Ca^2+^ ATPase
SGK1: serum/glucocorticoid-induced kinase
T_a_: ambient temperature
T_b_: body temperature
VLED: very low energy diet.
